# Connecting Neurons to a Mobile Robot: An In Vitro Bidirectional Neural Interface

**DOI:** 10.1155/2007/12725

**Published:** 2007-07-30

**Authors:** A. Novellino, P. D'Angelo, L. Cozzi, M. Chiappalone, V. Sanguineti, S. Martinoia

**Affiliations:** ^1^Neuroengineering and Bio-nanotechnology Group, Department of Biophysical and Electronic Engineering (DIBE), University of Genova, Via Opera Pia 11a, 16145 Genova, Italy; ^2^NeuroLab, Department of Informatics Systems and Telematics (DIST), Via Opera Pia 13, 16145 Genova, Italy; ^3^Center for Neuroscience and Neuroengineering “Massimo Grattarola”, University of Genova, 16132 Genova,Viale Benedetto XV, 3, Italy

## Abstract

One of the key properties of intelligent behaviors is the capability to learn and adapt to changing environmental conditions. These features are the result of the continuous and intense interaction of the brain with the external world, mediated by the body. For this reason “embodiment” represents an innovative and very suitable experimental paradigm when studying the neural processes underlying learning new behaviors and adapting to unpredicted situations. To this purpose, we developed a novel bidirectional neural interface. We interconnected in vitro neurons, extracted from rat embryos and plated on a microelectrode array (MEA), to external devices, thus allowing real-time closed-loop interaction. The novelty of this experimental approach entails the necessity to explore different computational schemes and experimental hypotheses. In this paper, we present an open, scalable architecture, which allows fast prototyping of different modules and where coding and decoding schemes and different experimental configurations can be tested. This hybrid system can be used for studying the computational properties and information coding in biological neuronal networks with far-reaching implications for the future development of advanced neuroprostheses.

## 1. INTRODUCTION

Electrophysiological techniques, both in vivo and in vitro, are traditionally used to study spontaneous neural activity and its modifications in response to different kinds of external stimuli (e.g., chemical, electrical, electromagnetic). One of the main limitations of these studies is the total absence of a sensory and motor “context.” This condition is particularly unnatural: complex mechanisms, like learning, are the result of a continuous interaction between the nervous system and the environment, mediated by the body. For this reason, during the last years, the growing interest in neuroscience for closed-loop experiments (cf. Society for Neuroscience Meeting 2004, San Diego (Calif, USA); http://apu.sfn.org) has led to the development of several innovative bidirectional platforms, under the hypothesis that the dynamical and adaptive properties of neural systems may be better understood in the context of the interaction between the brain and the external environment.

In the last few years, interaction has been studied at different levels of investigation: at the molecular level, by synthesizing the behavior of artificial ion channels—the dynamic-clamp technique (Sharp et al. [2]); at the single neuron level, by interfacing artificial and actual neurons (Le Masson et al. [3]); at the population level, by controlling the dynamic regime of neuronal populations (Wagenaar et al. [4]) and its adaptive properties (Shahaf and Marom [5]; Marom and Eytan [6]); and, finally, at the whole system level, by means of experiments in which portions of the ex vivo/in vivo brain of an animal are connected to artificial/virtual robots to form bioartificial/hybrid systems (Reger et al. [7]; Wessberg et al. [8]; Nicolelis [9]; Schwartz et al. [10]; Karniel et al. [11]).

An alternative and simplified paradigm to study the interaction between the brain and the external world is the “embodied electrophysiology,” where dissociated neuronal networks are bidirectionally coupled to artificial systems (DeMarse et al. [12]; Bakkum et al. [13]; Martinoia et al. [14]), which provide a physical body to the in vitro brain and allow it to interact with the environment (Potter [15]). This paradigm can be used to investigate the mechanisms that the nervous system uses to represent, store, and process sensory-motor information, understanding how living neurons lead to higher-level cognition and “intelligent behavior” (Bakkum et al. [13]).

The development of in vitro bidirectional neural interface offers the unique opportunity to explore the adaptive properties of a model of the neural system and it can be of valuable help for the future developments of in vivo neural interfaces (Mussa-Ivaldi and Miller [16]; Nicolelis [17]). Ideally, in vivo brain-machine interfaces should enable two-way communication, that is, both stimulation and recording at the same time. Two-way interaction would be particularly crucial in advanced neuroprostheses. Sensory systems cannot be fully restored by simply mapping input into the brain; instead, neuroprosthetic devices should be fused with the reciprocating neural interactivity that is responsible for ongoing conscious awareness.

**Figure 1:**
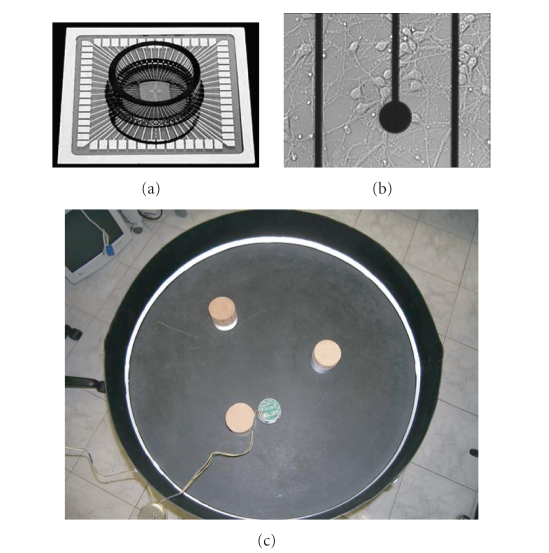
The main actors of the neurorobotic set-up. (a) A commercial MEA by Multichannel Systems (Reutlingen, Germany) with 60 electrodes. (b) Cortical neurons grow and develop a 2D network over the MEA, in proximity of a recording microelectrode. (c) The Kephera robot wandering in its arena, filled with cylindrical wooden obstacles.

The aim of this paper is to describe the architecture and the high potential of the developed neurorobotic system, that is, a neuronal network connected to a mobile robot. In the “methods” section, we discuss the issues underlying design and computational choices. The computational requirements for the closed-loop system are very demanding, mainly due to the necessity to simultaneously process high-frequency multichannel data, in real time. On the other hand, the novelty of this approach involves the necessity to explore different computational schemes (e.g., to change the coding/decoding strategy, the number of input/output electrodes, and the value of the parameters). In the “results” section, we describe the computational performances of the developed system and the strategies for selecting the input and output sites, an essential step when dealing with neuronal model with a no predefined architecture, such as dissociated cultures (see Figures Figure1(a), Figure1(b)). Finally, the preliminary experiments involving a network of cortical neurons and a robotic body are presented and the main improvements with respect to our previous results (Cozzi et al. [18]) are underlined, both in terms of computational architecture and experimental protocol. The use of a simple reactive behavior (i.e., obstacle avoidance) demonstrates the feasibility of the approach and the potential of this novel experimental paradigm.

## 2. MATERIALS AND METHODS

### 2.1. Robot body, playground arena and obstacle avoidance task

Modeling of adaptive behavior by developing adaptive autonomous agents is an approach widely investigated in the fields of artificial intelligence and autonomous control (Maes [19]; Brooks [20]), and a particular model of adaptive behavior is represented by an agent who is motivated in trying to survive in a defined environment, without any external (i.e., human) help.

The agent may generate its actions exclusively from the available sensory information, or may use some kind of previous “experience.” The former type of agent is generally referred as “reactive.” One of the most studied implementations of this model is the “exploring” vehicle paradigm. In 1984, Braitenberg [21] proposed a simple architecture, that is, a vehicle with direct links between sensors and motors (the greater the sensors values are, the faster the motors run), that seems to mimic an intelligent behavior in a real context. The easiest example of the Braitenberg's vehicles is a two-wheeled robot with two light sensors that, according to the connection between sensors and motor-wheels, can produce different and interesting behaviors (fear, aggressiveness). Here we will show a neurorobotic Braitenberg “explorer” vehicle as an example of application of the closed-loop platform for embodied electrophysiology.

The artificial body consists of a small mobile robot (Khepera II, K-team, http://www.k-team.com), equipped with two wheels and eight infrared (IR) proximity sensors that provide information about the distance of the robot from obstacles. In our experiments, the robot (7 cm diameter) moves inside a circular arena (80 cm diameter), containing wooden cylindrical obstacles (7 cm diameter). The Khepera robot and its playground are shown in Figure 1(c). In order to partially compensate the high nonlinearity of the proximity sensors and the influence of the ambient light, the internal perimeter of the playground and the border of the obstacles were covered with an IR reflective tape.

### 2.2. Computational architecture of the neural interface

**Figure 2:**
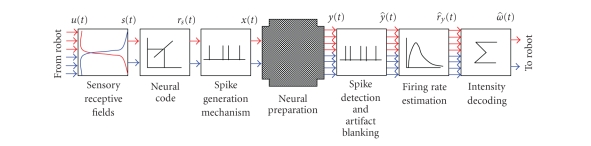
Computational architecture of the closed-loop system. The signals coming from the infrared sensors (IR) of the robot are translated into patterns of stimuli that are delivered to the neural preparation through a set of selected stimulating electrodes. Then the activity recorded by two groups of electrodes is evaluated in terms of firing rate (i.e., mean number of detected spikes/s) and used as driving speed for each of the robot's wheel.

To establish a bidirectional communication between the neuronal preparation and a mobile robot, the electrophysiological signals need to be translated into motor commands for the robot (decoding of neural activity), and at the same time the sensory signal from the robot need to be translated into a pattern of electrical stimulation (coding of sensory information). Figure 2 presents the general computational architecture of the proposed closed-loop system that can be summarized in the following three main parts (i.e., from left to right in Figure 2).

1. Coding (from the robot to the neural preparation): while the robot freely moves into the playground, its IR sensors see whether or not an obstacle is in the proximity and where it is (left or right side). The IR signals *u*(*t*) are weighted according to the sensory receptive field law and the two resulting stimulation signals *s*(*t*), relative to the right and left “eye” of the robot, are then coded into a feedback stimulation *x*(*t*)
2. Processing of electrophysiological signals: the spontaneous or evoked electrophysiological activity *y*(*t*) is sampled $\left(\hat{y}(t)\right)$ and processed, in order to give an estimation of the instantaneous firing rate ${\hat{r}}_{y}(t)$
3. Decoding (from the neural preparation to the robot): the processed electrophysiological signal ${\hat{r}}_{y}(t)$ is translated into motor commands *ω*(*t* for the robot, according to the specified decoding strategy.

To make our computational architecture as open as possible, we developed a library of coding/decoding schemes (Cozzi et al. [18]; Cozzi et al. [22]), and identified the most effective ones in achieving a desired behavioral task.

**Library of coding schemes**

Coding means the representation of external sensory input patterns in terms of electrical stimulation and hence of neuron's activity. In this perspective, the implemented *neural code* has been of two main typologies, both of them based on the rate coding concept.

1. Proportional coding.
  The rate of stimulation *r*_s_(*t*) is proportional to the sensory feedback *s*(*t*). The maximum rate of stimulation, *r*_*s*_^*max*^⁡, is only attained when the robot hits an obstacle. It was suggested that this value has to be as large as possible for accurate coding of the temporal structure of sensory signal (Wagenaar et al. [1], DeMarse et al. [12]), but at the same time it has to be low in order not to damage the culture (Shahaf and Marom [5]), therefore, the maximum of the stimulation rate *r* *_s_*(*t*) was up to 2 Hz.
2. Binary coding.
  A binary coding scheme generates trigger signals for the electrical stimulator only when the sensory feedback *s*(*t*) overcomes a threshold, approximately reflecting the presence of an obstacle at 5 cm distance. The stimulation rate *r*_*s*_(*t*) is therefore either 0 or 1 Hz. The maximum frequency of stimulation was chosen according to what reported in literature (Shahaf and Marom [5]).

**Preprocessing of electrophysiological signal**

*Spike detection* . The electrophysiological signals $\hat{y}(t)$ acquired from MEA electrodes must be preprocessed in order to remove the stimulus artifact and to isolate spikes from noise.

The spike detection algorithm uses a differential peak-to-peak threshold to follow the variability of the signal (Grattarola et al. [23]). A time window, sized to contain at most one single spike (4 ms), is shifted along the signal, sampled at the frequency of 10 kHz. Within the window when the difference between the maximum and the minimum exceeds the threshold, a spike is found and its time stamp is saved. In this way, the resulting spike train signal is sampled at 250 Hz. The threshold is proportional to the noise standard deviation (SD) and is calculated separately for each individual channel (typically as 7 × SD) before the beginning of the actual experiment (i.e., the spontaneous activity recording, see Section 3.3).

*Blanking of stimulus artifact* . Stimulus artifacts are detected when the recorded signal exceeds a second, higher threshold. The artifact is then suppressed by cancelling the first sample in the spike train occurring immediately after it, corresponding to a signal blanking of 4 milliseconds after stimulus delivery. This quite conservative procedure could have been improved, but we found it effective for our applications.

**Decoding schemes**

Although several linear and nonlinear algorithms for translating neuronal activity into motor commands for external actuators have been proposed (Chapin et al. [24]; Wessberg et al. [8]; Carmena et al. [25]; Wessberg and Nicolelis [26]), here the decoding schemes are simply based on rate-coding (Fetz [27]), that has proven to be efficient in brain-machine-interfaces (Lebedev and Nicolelis [28]).

*Firing rate estimation.* The neural activity is represented by the instantaneous firing rate ${\hat{r}}_{y}(t)$ on each recording channel and it is estimated from the spike trains *y*(*t*) through a low-pass filter. Two different filters have been implemented (a first-order and a second-order filter).

*Decoding.* The recording sites are divided into two groups, respectively used for controlling the left and right wheel, each of them formed by *N* electrodes. The motor commands *ω*(*t*), that is, the angular speeds of the wheels, are obtained by implementing the following winner-takes-all (WTA) mechanism:

(1) $$\begin{array}{ll} \omega_{L}(t) & =\{\begin{array}{cc} \left(\omega_{0}-\sum_{i=1}^{N}C_{i}\cdot {\left[{\hat{r}}_{i}(t)\right]}_{R}\right) & \text{if}\omega_{L}\geq \omega_{R},\\ -\omega_{b} & \text{if}\omega_{L}<\omega_{R}, \end{array}\\ \omega_{R}(t) & =\{\begin{array}{cc} \left(\omega_{0}-\sum_{i=1}^{N}C_{i}\cdot {\left[{\hat{r}}_{i}(t)\right]}_{L}\right) & \text{if}\omega_{R}\geq \omega_{L},\\ -\omega_{b} & \text{if}\omega_{R}<\omega_{L}, \end{array} \end{array}$$

where *ω*_*b*_ is a constant angular speed (up to 2 rad/s), *ω*_*0*_ is the maximum angular speed (i.e., 5 rad/s); ${\hat{r}}_{i}(t)$ is the instantaneous firing rate of the recording site *i*, *C* *_i_* is a normalization coefficient. *L* and *R* indicate signals pertaining respectively to the left and the right wheel. In absence of neuronal activity, the robot goes straight with a constant angular speed of 5 rad/s that corresponds to a linear velocity of 16 cm/s. The coefficients *C*_*i*_ can be computed according to different criteria: they represent an estimate of the strength of the connection between the corresponding input and output site (computed by means of a linear regression), as reported in (Cozzi et al. [18]), or they simply represent the inverse of the estimation of the maximum value that can be reached by the instantaneous firing rate on each group (left versus right) of electrodes. The first method is usually applied when decoding the activity of each unit in large population of neurons (Georgopoulos et al. [29]). For the experiments reported here, we adopted the second: the used algorithm already selects input-output pathways characterized by the maximal strength of the functional connection and we only need to equalize them, in other words, when the robot is far from obstacles the spontaneous activity should not cause the robot turning preferentially clockwise or counterclockwise. Assuming that neurons on the left and right sides are mostly excitatory, the minus sign in the control law allows us to implement inhibitory contralateral connections between inputs and outputs.

The WTA strategy has proven to be a more appropriate decoding scheme than those already presented in our previous work (Cozzi et al. [18]). In fact, even though the WTA mechanism may result in movements that are less smooth, the lowest values chosen for the angular speed (i.e., 2 or 5 rad/s instead of 10 rad/s) facilitate the robot rotation without affecting the general behavior and, as a consequence, the robot can reverse direction in much less space, actually realizing what the “brain” is ordering to its “body.” This strategy is also suggested by the nonlinearity of the IR sensors (for further details see the “Khepera II-IR sensors report,” http://ftp.k-team.com/khepera/documentation/Kh2IRAN.pdf) of the robot that are capable to reliably detect an obstacle only when the robot is closer than about 5 cm from an obstacle.

### 2.3. Neural preparation and control architecture

**Neural preparation and electrophysiological set-up**

Dissociated neurons in culture randomly rearrange in a bidimensional structure and, once they have established synaptic connections, they show spontaneous neural activity (starting from about 7 days in vitro DIVs) that can be modulated by means of electrical stimulation (Maeda et al. [30]; Jimbo et al. [31]; Marom and Shahaf [32]). We used dissociated cultures of cortical neurons, extracted from rat embryos (E18). Using standard methods previously described (Martinoia et al. [33]; Chiappalone et al. [34]), cells were plated on microelectrode arrays (MEAs) (Figures 1(a), 1(b)) with 60 TiN/SiN electrodes (diameter 30 *μ*m, interdistance 200 *μ*m) arranged on an 8 × 8 square grid. Experiments were performed in the range 18–42 DIVs, when the neuronal network reaches its “mature” state, consisting of synchronized clustered activity with minute-to-minute fluctuations in the probability of firing (Marom and Shahaf [32]).

The experimental set-up is based on the MEA60 system supplied by Multi Channel Systems (MCS, Reutlingen, Germany). The system is constituted by the following elements: a neuronal preparation cultured over an MEA, a mounting support with a 60-channel amplifier (gain 1200x), a home made 8-channel stimulus generator, to deliver both current and voltage desired stimulating signals, an inverted optical microscope, connected to a CCD camera (Hamamatsu, Japan), to visually monitor the cells during the experiment, an antivibration table and a Faraday cage.

Raw data are also monitored and recorded by using the commercial software MCRack (Multi ChannelSystems, Reutlingen, Germany) (sampling frequency was set to 10 kHz/channel). To confirm real-time behaviour, neural data were also processed offline by using ad-hoc developed software tools (Vato et al. [35]; Chiappalone et al. [34]).

**Control architecture**

The control architecture presented in our previous work (Cozzi et al. [18]) has partially changed and some feature has been added. In particular, we have moved from xPC-Target (http://www.mathworks.com/products/xpctarget) that was not able to handle and log the very large amount of data coming from neuronal network, to the QNX 6.1 (QNX software systems), a POSIX-compatible operating system specific for hard real-time applications.

The present architecture involves three PCs. PC1 (P4, 2.8 GHz, 512 MB RAM), that is, the one that runs QNX, is equipped with an A/D board PCI-6071E (National Instruments, Texas, USA) and it is responsible for (a) electrophysiological signals acquisition, (b) online spike detection and artifact blanking, (c) decoding of the spike trains, (d) handling the serial communication with the robot and PC3, (e) coding of robot's proximity sensors signals, (f) production of the pattern of stimuli that trigger the electrical stimulator, and (g) experimental data logging (spikes, sensors activity, wheel speeds, stimuli, robot trajectory). These tasks are processed by different threads at different sampling rates, in particular tasks (a)-(b) are at 10 kHz, tasks (c)–(e)-(f)-(g) are at 250 Hz, and task (d) is at 10 Hz.

A second computer, PC2 (P4, 2.8 GHz, 512 MB RAM, Win2000), connected to PC1 through an Ethernet link, is the experimental front-end. We used Simulink/Real-Time Workshop (the MathWorks) and the RT-Lab package (Opal-RT) as development environments. This package generates two processes that are executed in real-time on the target node, that is, PC1. This system allows simultaneous acquisition of neural signals from up to 32 recording sites.

PC3 (P4 2.8 GHz 512 MB RAM, QNX 6.1) is in charge of real-time tracking of the robot movement and it is connected through a serial cable to the main computational node of the architecture (PC1). A CCD camera (DSE TCC5 with 1/3 CCD SONY SuperHAD) is placed 1.5 m above the central position of the arena. This physical arrangement of the camera allows a good resolution while minimizing distortion at the boundaries of the arena. A frame-grabber (Arvoo Picasso PCI-2SQ) acquires samples at 5 Hz from the camera with a resolution of 640 × 480 pixels. One pixel on the CCD sensor corresponds to ∼ 2 mm, so that the arena is contained in a 400 × 400 subwindow at the center of the image. The detection phase is performed in a small square portion (50 × 50 pixels) of the global field of the CCD camera (the detection in such small area is low demanding in computing performance and thus the process can be performed in real-time). This square area represents the predicted robot location. A red round marker placed onto the top of the robot and the detection of its position is based on a local evaluation of the intensity of the RGB values of every pixel belonging to the detection window.

### 2.4. Data analysis

Processing of electrophysiological signals

*Poststimulus Time Histogram* . To investigate the neural activity evoked by stimulation, we computed the poststimulus time histogram (i.e., PSTH), which represents the impulse response of each site of the neural preparation to electrical stimulation. The PSTHs were calculated by taking 400 ms time windows from the recordings that follow each stimulus. We then counted the number of spikes occurring in a 2–4 msec bin and divided this measure by the number of stimuli (Rieke et al. [36]). For our cultures, typical PSTHs show an “early” (less than 50 msec) and a “late” (50–250 milliseconds) component (Shahaf and Marom [5]; Marom and Shahaf [32]; Cozzi et al. [22]).

*Stimulus-Triggered Speed* . The stimulus-triggered speed (STS) is constructed by averaging the speed waveform due to each stimulus. The robot has two independent wheels, whose speeds are proportional to the neuronal activity of two “brain” regions (i.e., the two set of electrodes selected as motor areas within the network). These regions receive sensory feedback by two independent stimulating sites. It is possible to construct two STSs for each input-output, that is, sensory-motor pathways, for a total of 4 curves (i.e., the variations of the speed of the left and the right wheels in response to the left stimulus are the first two curves and constitute the first STS, and the variation of the speed of the left and the right wheels in response to the right stimulus constitute the second STS with the latter two curves), and it is also possible to study the performance of the robot behavior by studying the side-selectivity of the relationship between stimuli and speeds.

**Indicators of robot performance**

The behavior of the neurorobotic system can be evaluated by means of the *stimulus-triggered speed (STS)* , that is, the average motor commands elicited by a single electrical stimulus:

(2) $$\text{STS}(\tau )={\langle \omega (\tau -t_{i})\rangle }_{i},$$

where *t* *_i_* is the time instant of delivery of the *i*th stimulus and *τ* is the time coordinate.

In order to have a more general evaluation of the robot performance during each trail (5′), we also used the following three parameters:

- *number of hits*,
- *trajectory length*,
- *space covered*. The percentage of the arena area covered by the robot path:
  (3) $$\text{SC}=\frac{\text{NP}\cdot cf^{2}}{A_{\text{arena}}-nA_{\text{obstacle}}}\cdot 100\%,$$
  where NP is the number of pixels covered by the robot, *c* *f* ^*2*^ is the area of one pixel, *n* is the number of obstacles in the playground, *A*_*arena*_ and *A*_*obstacle*_ are, respectively, the areas of the playground and of each obstacle.

The software tools for of-line signal processing aimed at the analysis of the behavior of the neurorobotic system were developed using Matlab 6.5 (the MathWorks).

## 3. RESULTS

### 3.1. Computing performance

**Figure 3:**
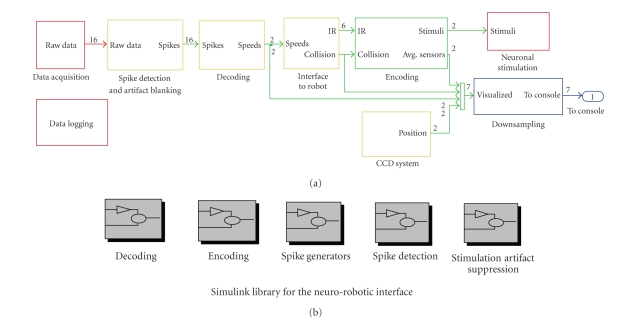
Computing performances in the Simulink implementation of the neurorobotic interface. Different colors correspond to different sampling rates (red = 10 kHz, green = 250 Hz, blue 5 Hz, yellow = mixed values), whereas the numbers indicate signal dimensions (i.e., in this case, we had 2 inputs and 16 outputs). The percentages in each block indicate the relative simulation time for each of the modules of the neurorobotic interface (2 inputs and 16 outputs). (b) Library of the modules that can be used in the neurorobotic interface. Each subset encloses Simulink blocks implementing different algorithms for that purpose.

The feedback loop computation time reached by our final neurorobotic architecture is under 1 millisecond; therefore, the real-time performance in the closed-loop system is compatible with the response time (4 ms) of our neuronal model. This value includes the time needed for (I) the electrophysiological signals acquisition, (II) the spike detection and the artifact suppression, (III) decoding of neural activity, (IV) computing of the speeds of the robot's wheels, and (V) coding of sensory feedback. The relative computational loads for each block are displayed in Figure 3(a): the most time-consuming parts are those running at 10 kHz, for technical reasons, and the blocks including sampling rate transitions, such as the interface with the robot, with the CCD camera system and with the stimulator. In these experiments, we used a robot with a standard RS232 interface that supports a baud rate of 9600 bit/s. We expect that by including a more recent protocol (e.g., USB2 or Firewire), the block would be less time consuming and would assure better performance. Spike detection and artifact blanking are also time-consuming due to the high dimension of the signals being processed. The performances were evaluated by means of Simulink Profiler, reported schematically in Figure 3(b).

### 3.2. Identification of input-output sites

In order to obtain a reactive behavior, we need the network to respond soon after the feedback stimulation, that is, we need input-output pathways characterized by a relatively early (up to 50 ms) and sustained response meaning a “high strength” in the functional connectivity (Shahaf and Marom [5]). If the network reacts to the sensory feedback and the evoked electrophysiological response is characterized by a relatively long activation phase (up to 200–300 ms), the robot would not be able to react to the presence of an obstacle in 100 ms (i.e., the delay among successive serial communications between the system and the robot). This is one of the reasons why we need to accurately select the input-output pathways, beside the fact that only low-frequency stimulation can be delivered for not fatiguing the culture (Shahaf and Marom [5]; Eytan et al. [37]). We need the stimulus-evoked response to be fast, prolonged, reliable, and therefore effective for the entire duration of the experiments (i.e., all day long).

As already said, the general aim is to have a robot that follows a specific task on the basis of the spontaneous/stimulated electrophysiological activity shown by the neuronal culture. To this end, it is a fundamental pre-requisite to characterize the collective activity of the network that will be connected to the robot (i.e., analysis of both spontaneous and stimulus evoked neuronal activity). This characterization phase is necessary since the unstructured nature of the culture does not allow us to a priori define the sensory and motor areas that will be connected with the sensory and motor areas of the robot, as it happens with portion of tissue with a well-defined sensorimotor architecture (Reger et al. [7]; Karniel et al. [11]).

Thus, the goal of the characterization phase is to select those channels of the MEA to be used as sensory inputs (i.e., connected to the robot's sensors) and motor outputs (i.e., connected to the robot's wheels) of the biological network.

To test the response to stimulation from different sites in different areas of the neuronal network, trains of 50 electrical stimuli are delivered (1.5 V peak to peak-extracellular stimulation, 500 *μ*s, and duty cycle 50%). This procedure is repeated from at least 5 arbitrarily chosen electrodes (Wagenaar et al. [38]).

The poststimulus time histogram-(PSTH) (i.e., the average number of spikes obtained in response to a stimulus, at different latencies) is then used for quantifying the strength of connections between a specific stimulating sites and all the other recording sites. It is the impulse response (in terms of instantaneous firing rate) to a single test stimulus.

The algorithm for the selection of the output (motor) and input (sensory) sites supplies the I/O pairs corresponding to maximum selectivity and it is based on network effective functional interconnectivity. The ideal case is described in the following: given two (or more) stimulating channels (e.g., S1 and S2) and two groups of recording sites (e.g., R1 and R2), the strength of the connectivity S1-R1 and S2-R2 is “high” and simultaneously, the strength of the connectivity S1-R2, and S2-R1 is “low” (i.e., good selectivity in input-output pairs). The described scheme guarantees, somehow, that the responses in the two (groups of) recording sites are different on the basis of the stimulating electrodes. Of course the above is an ideal situation and, since the mean connectivity of the network is high, also due to the high density of plated cells, it is hard to get highly specific responses in the input-output pathways.

The methodology that we developed to make a selection of the pathways is the “selectivity map” (see Section 3.3 for a typical map). Each dot represents the PSTH area at a specific recording site given that there was a stimulation from a couple of stimulating sites. All the possible input-output combinations are explored and only the pathways producing responses lying more distant from the diagonal (i.e., closer to the axis) are selected.

Those specific pathways (of sensory-motor activations) can be then conveniently utilized for driving the robot and for implementing simple reactive behaviors (e.g., obstacle avoidance), as presented in Section 3.1.

### 3.3. Example behaviors of the neurorobotic system

Once the role of the microelectrodes (i.e., selection of input-output sites) has been decided, the experiment can start. A desired result is achieved when an improvement of the robot behavior is confirmed by possible modification of the neuronal network dynamics (i.e., adaptation).

Each experiment with the neurorobotic system is usually divided into the following phases:

- spontaneous activity recording (5 minutes);
- preprocessing: test stimulus from 8 channels (serial stimulation);
- input-output channel selection: at least 2 channels for input (sensors) and 2 channels for output (motors).
- closed–loop experiment: Robot running (5 + 5 + 5 minutes):
  - free running;
  - obstacle avoidance with the application of a learning protocol (when the robot hits an obstacle, a conditioning stimulus at 20 Hz frequency is delivered from the collision side). The learning protocol is based on what reported in literature: only a few examples of learning (i.e., potentiation and depression) have been demonstrated for dissociated neurons cultured over MEAs and all of them are based on the application of trains of stimuli at “high” frequency (Jimbo et al. [39]; Jimbo et al. [40]; Tateno and Jimbo [41]; Bonifazi et al. [42]; Ruaro et al. [43]). We have evidence that similar protocols have the effects to mainly potentiate the network electrophysiological response in terms of number of evoked spikes (Chiappalone et al. [44]);
  - free running.
- post-processing:
  - spontaneous activity recording (5′);
  - test stimulus from the two chosen stimulating channels.

To avoid manual removal of the robot and possible damage due to wheels' motors heating in case of a collision against an obstacle, a step-back mechanism (2 seconds backward movement with an angular speed of 5 rad/s) was implemented.

A user-friendly GUI allows (i) to select input and output channels (i.e., recording and stimulation sites), (ii) to choose among different coding and decoding strategies, and (iii) to change all the experimental parameters (e.g., spike-detection threshold, maximum robot speed, cut-off frequency of the filter for the estimation of neural activity, maximum stimulation rate).

**Figure 4:**
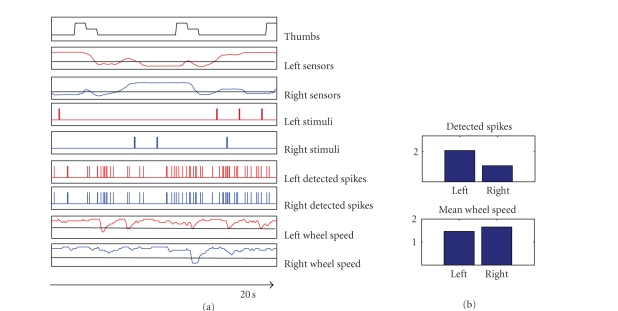
Signals obtained at different levels of the neurorobotic interface during 20 seconds of a free running session (c.f. Results, for a detailed description of the experimental protocol). In this particular experiment we used 2 stimulation sites and 2 recording sites. When the robot is approaching an obstacle, the value of sensors increases and when it overcomes a threshold (i.e., 500 levels) a feedback stimulus is delivered (max frequency 1 Hz). The left and right detected spike trains are then processed into motor commands, that is, left and right wheel speeds (the line corresponds to 0 rad/s). The network was spontaneously active and during this phase we recorded 2046 spikes from left and 1057 from the right one, resulting in a mean wheel speed of 1.4 rad/s (left) and 1.6 rad/s (right).

The signals obtained at different levels in the bi-directional interface are represented in Figure 4. The spike trains are then low-pass filtered to obtain instantaneous firing rates that are considered as indicators of the level of neural activity. The cutoff frequency of the filter is set, in this case, at 1 Hz. The values corresponding to reasonable response times range from 0.5 to 1 Hz: in fact they represent a good compromise between fast response and time integration requirements. The previously adopted cutoff frequency of 0.1 Hz (Cozzi et al. [18]) was not suitable for bursting networks because the long-term effect of time integration lets the past events (previous bursts) to weight more than instantaneous activity.

The motor commands (i.e., the speeds) are then obtained according to the control law which implements inhibitory contro-lateral connections between inputs and outputs (see decoding schemes section for details), thus we expect that a feedback stimulus coming from left sensors would result in a decrease of the speed of the right wheel of the robot, and a stimulus coming from right sensors would determine a deceleration of the speed of the robot's left wheel. The proximity signals coming from the sensors placed on the two sides of the robot are averaged in order to obtain two feedback signals, each of them related to one side of the robot. Figure 4 shows the patterns of stimuli obtained from the feedback signals, according to the binary coding scheme.

A number of preliminary experiments were performed using, respectively, 2 stimulation and 2 recording sites. In the following, the results of two example experiments are reported to let the reader better understand the experimental procedure and appreciate the performances of the developed closed-loop system.

**Figure 5:**
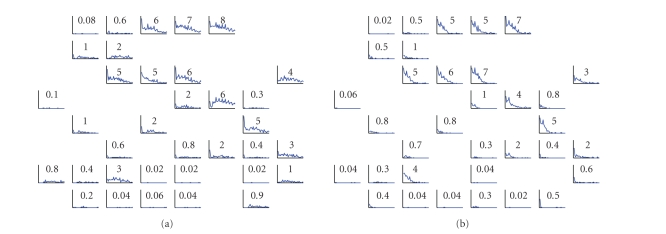
PSTHs of the evoked responses in a cortical neuronal network. (a) The post stimulus time histograms obtained in all the responding channels are reported over an 8 × 8 grid (i.e., reproducing the layout of the MEA) after the stimulation from site 46 —fourth column, sixth row. The small number reported in each box represents the area of the histogram. Not responding channels are excluded. (b) Responses evoked in the network by stimulation from site 62. As it can be clearly notices the channels responding to site 46 respond also to channel 62, denoting an absence of strong selection with respect to the stimulating electrode. X-scale [ 0, 1 ]; Y-scale [ 0, 400 ] milliseconds.

Figure 5 shows the PSTHs obtained during the characterization phase of one example experiment. The responses evoked from different stimulation sites are similar (i.e., Figures 5(a) and 5(b)), thus revealing a low degree of selectivity and a high degree of connectivity. In a case like the one presented in Figure 5, the preparation can hardly be used to control the robot and it is discarded.

**Figure 6:**
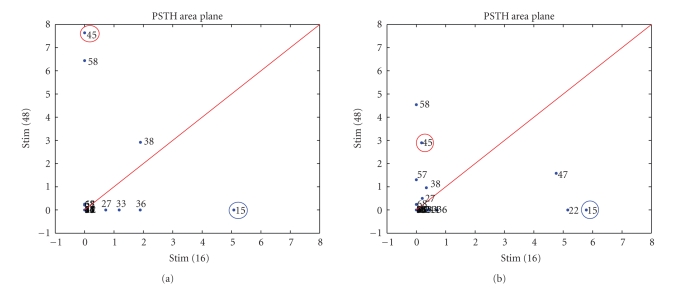
Connectivity maps (same data reported in Figure 4). The connectivity map represents a plot of the PSTH areas evoked by a couple of stimulating electrodes on a specific electrode. In this way we are able to represent the network response to a specific choice of stimulating sites. The ideal case should be to have two recording electrodes placed on the axis, far from the origin (i.e., maximum response to one stimulating electrode and zero to the other). (a) Before the robotic experiment. (b) After the robotic experiment.

Figure 6 presents an example of good connectivity maps obtained during the characterization phase (Figure 6(a)) and after the robotic experiment (Figure 6(b)): the electrodes 15 and 45, that will be further chosen as recording electrodes, are positioned close to the axis, indicating that their responses to the stimulating channel are selective (see Section 3.2 for further details).

**Figure 7:**
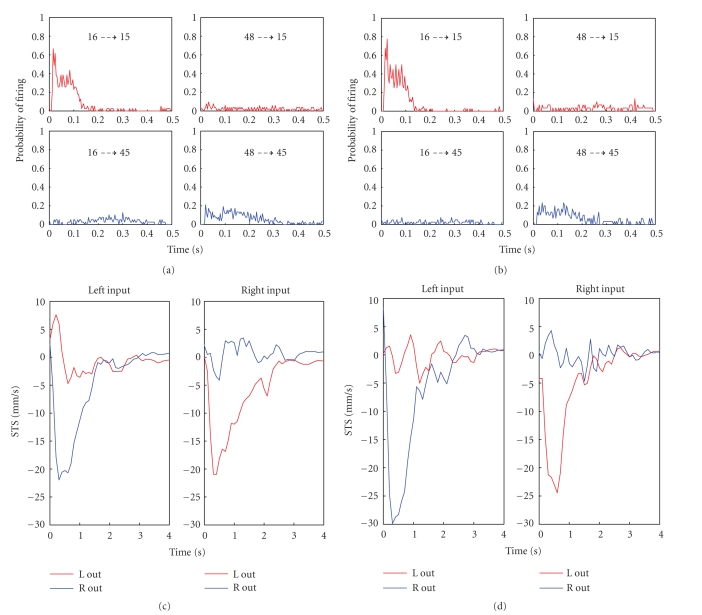
PSTH and STS in a neurorobotic experiment. (a) PSTHs for two electrodes (chosen as recording —motor electrodes) with respect to two stimulating sites. Electrode 15 responds well to stimulation from electrode 16 and bad to stimulation from electrode 48; on the contrary electrode 45 responds well to 48 and bad to 16. This tendency is maintained and even improved after the robotic experiment (b). The STS graphs before (c) and after (d) the robotic experiment prove again the selectivity of the chosen electrodes and the improvement in the performances (increased STS area).

Figures Figure7(a) and Figure7(b) show the PSTHs corresponding to the inputs/outputs chosen after the characterization phase; during an experimental session with the robot (i.e., experiment is different from the previous one). In this example, the recording electrode 15 is very sensitive to the stimulation delivered from electrode 16 (top left) while it is quite unaffected by stimuli delivered form electrode 48 (top right). At the same time, the recording site 45 is not sensitive to stimuli coming from electrode 16 (bottom left) while it is very affected by stimulation from electrode 48. In this case, different stimulation sites evoke very different response, thus revealing a high degree of selectivity that is also confirmed by the connectivity maps presented in Figure 6.

The shapes of the PSTHs must be similar to those of the PSTHs obtained during the characterization phase in order to ensure the stability of the response of the neuronal culture. If the area of the PSTHs drastically decreases at the end of the closed loop phases it means that the neuronal network has been fatigued by excessive repeated stimulations (Shahaf and Marom [5]). The wellness and stability of the culture are “sine qua non” conditions to be verified before describing the neurorobotic behavior by means of the robot's performance indicators. Under these conditions, the performance of the neural preparation in controlling the robot can be represented by the STSs, depicted in Figures Figure 7(c) and Figure 7(d).

**Figure 8:**
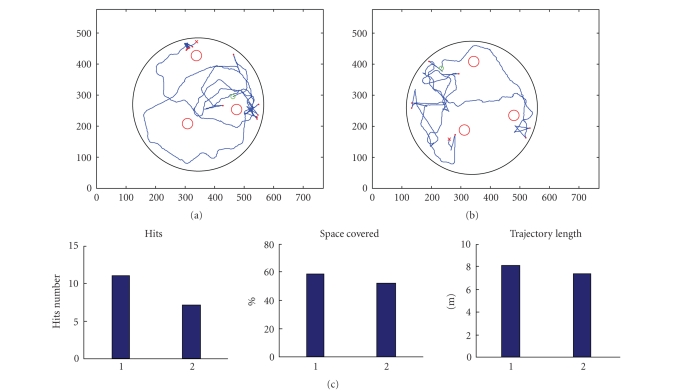
Robot trajectories and performances in a neurorobotic experiment. (a) Robot trajectory during the first free running phase. (b) Robot trajectory during the last free running phase (i.e., after the learning phase). (c) Indicators of the robot's performance. The last two parameters only show that the two phases are comparable in terms of covered space and trajectory length during the robot's movement inside the arena. For this reason, the reduction of hits in the second phase (i.e., first parameter) suggests an improvement of performances during the obstacle-avoidance task. The conclusion is that an improvement in the robot's behavior in terms of a decreased number of hits must depend from the modulation of the neuronal activity, as also confirmed by the graphs of the STS presented in the previous figure.

Examples of the robot trajectories are presented in Figures Figure 8(a) and Figure 8(b). Figure 8(c) shows the indicators generally used for quantifying the robot performance. The first indicator alone, that is, the number of hits, is not sufficient for describing the performances of an obstacle avoidance task. In fact, a low number of hits could result from limited robot movements or from the repetition of the same trajectory. For this reason, it is necessary to consider also the fraction of space covered by the robot and the length of its trajectory. Together, these simple indicators evaluate the robot performances inside the arena, even if they are not related to the sensory feedback coming from the external environment. These parameters do not allow quantifying any relationship between the motor response and the sensory information, but, considering different phases, if the robot covered the same area and the trajectories are in the same order, then the two phases are comparable, and a reduction of the number of hits should indicate an improvement of the robot's behavior. An improvement in the robot's behavior must correspond to an improvement in the relationship between the motor response and the feedback sensory information (i.e., the STSs). The STS is the only parameter that permits to understand and demonstrate whether a different behavior of the robot actually corresponds to a different dynamics of the neuronal activity, and for this reason it can be considered the best indicator of the performance of the overall neurorobotic system.

The comparison of the STSs and connectivity maps obtained during each phase illustrates that a modification in the robot's behavior has occurred. Therefore, one could speculate that the origin of such a modification relies on specific synaptic changes, (i.e., Hebbian potentiation in terms of number of evokes spikes) of the neurons placed at the recording electrodes (Jimbo et al.[39]; (Jimbo et al. [40]; Tateno and Jimbo [41]; Bonifazi et al. [42]; Ruaro et al. [43]). We cannot infer or demonstrate that synaptic changes are pathways specific but considering the global behavior of the recording sites after a neurorobotic experiments a possible effect at (sub)population level (i.e., a kind of network plasticity) has occurred induced by the external correlated stimulation (Chiappalone et al. [44]).

## 4. DISCUSSION AND CONCLUSION

“We have this common internal neural language that we are born with and so if you can exploit that with the right stimuli then you are going to help the brain develop to do the things like reason.” (Shaw [45].)

We have developed a general real-time, bidirectional neural interface platform. The system is capable of acquiring multisite electrophysiological activity at 10 kHz per channel, to perform spike detection and artifact suppression, from up to 32 channels. That is a step forward with respect to simpler systems (Reger et al.[7]; Karniel et al.[11]) or to bidirectional systems implemented by others (DeMarse et al.[12]; Bakkum et al. [13]) or by ourselves (Martinoia et al. [14]; Cozzi et al.[18]).

The use of portions of brain, such as the brainstem of a sea lamprey, connected to an artificial device represents the very first application of the “embodied electrophysiology” paradigm. The main difference between the lamprey-based neurorobotic system and the bidirectional interface we developed is the fact that in the lamprey preparation the circuitry governing the stabilization and orientation during swimming (Deliagina [46]; Deliagina et al. [47]) maintains the original citoarchitecture and the natural input-output system is used as a controller to drive the robotic body (Reger et al. [7]; Karniel et al. [11]).

Potter and colleagues (DeMarse et al. [12]; Bakkum et al. [13]) overcame the simplification of an already structured portion of brain presenting an innovative embodied electrophysiology paradigm in which a randomly grown neuronal networks controlled a simulated body. As they reported, it was simply a “neuroethology experiment” to merely observe the effect of feedback stimulation on the general behavior and where their “animat” has not to perform any particular task. Our neurorobotic interface, on the contrary, has to work in a reactive manner expressing a kind of “stimulus-driven behavior.” Here we reported, in details, the methodology of the system (including hardware and software features), an optimized way to identify the “functional I/O pathways” and we presented a method to analyze the robot behavior and correlate it to the network electrophysiology.

As described in the previous sections, in our system, the spike trains can be decoded into motor commands (at 250 Hz) through a variety of decoding strategies. Such motor commands are then used to control a mobile robot, to which a specific task is assigned. Conversely, sensory signals can be coded into patterns of stimulation (again, according to a variety of coding schemes) and sent to a custom electrical stimulator with up to 8 stimulation channels. Although the algorithms used for spike detection and artifact blanking are simple, compared to those adopted in other experimental frameworks (Wagenaar and Potter [48]; Obeid and Wolf [49]), they allow a good level of reliability with the advantage of an extremely light computational load. It should be underlined that the presented experimental paradigm can be extended to other computational schemes and one of the key features of the system is to allow testing different coding and decoding strategies in relationship with optimal coding and performances and with the capability of the neuronal system to adapt for a new task in an actual closed loop environment.

The software architecture is flexible and modular, and allows fast prototyping of new modules according to the experimental requirements. Real-time performance was very good and it is comparable to other simpler systems (in terms of recording/stimulating channels), previously described in the literature (Reger et al. [7]; Martinoia et al. [14]; Wagenaar and Potter [38]; Cozzi et al. [18]; Karniel et al. [11]; Wagenaar et al. [50]). We developed a library of coding and decoding modules, which, as mentioned, could be easily extended. This is a key point with respect to possible implication for the development of novel brain-machine interface with enhanced capabilities and bidirectional interactivity.

Concerning our particular application, we also developed the tools for studying the ability of a culture of cortical neurons to process information in order to drive a robot according to a defined motor task (with a particular emphasis on the method for input-output pathways selection), and at the same time it allows to supervise the population activity changes in response to external feedbacks. It should be stressed that this is the first time that a closed-loop neurorobotic system (with in vitro neuronal populations) is utilized for performing specific behavioral oriented tasks.

The proposed experimental framework creates new possibility for investigating basic mechanisms of learning and adaptation (e.g., distributed synaptic plasticity, long term potentiation (LTP) and long term depression (LTD)) by directly studying how behavior arises from the emerging collective dynamics of a neuronal ensemble. Additionally, the experimental system could be also conveniently utilized and adapted to other in vitro models such as acute, organotypic slices, and patterned neurons, where the network architecture is partly preserved or can be designed.

Finally, on a long term perspective, this approach could have a relevant impact in the field of bio-inspired computational systems and for the development of novel brain-computer interfaces and of advanced neuroprosthetic devices.
